# Simvastatin-induced breast cancer cell death and deactivation of PI3K/Akt and MAPK/ERK signalling are reversed by metabolic products of the mevalonate pathway

**DOI:** 10.18632/oncotarget.6304

**Published:** 2015-11-11

**Authors:** Tingting Wang, Serena Seah, Xinyi Loh, Ching-Wan Chan, Mikael Hartman, Boon-Cher Goh, Soo-Chin Lee

**Affiliations:** ^1^ Cancer Science Institute of Singapore, National University of Singapore, Singapore, Singapore; ^2^ Department of Haematology and Oncology, National University Cancer Institute, National University Health System, Singapore, Singapore; ^3^ Department of Surgery, National University Cancer Institute, National University Health System, Singapore, Singapore

**Keywords:** simvastatin, PI3K/Akt/mTOR, MAPK/ERK, mevalonate pathway, breast cancer

## Abstract

Statins purportedly exert anti-tumoral effects on breast cancer. However, the biologic mechanisms for these actions are not fully elucidated. The aims of this study were 1) to explore the effects of simvastatin on apoptosis, proliferation as well as PI3K/Akt/mTOR and MAPK/ERK pathway in a window-of-opportunity breast cancer trial; 2) to further confirm findings from the clinical trial by functional studies; 3) to explore the regulatory role of mevalonate pathway on the anti-tumoral effects of simvastatin. In clinical samples, simvastatin led to increase in cleaved caspase-3 (*p* = 0.002) and decreased trend for Ki67 (*p* = 0.245). Simvastatin markedly suppressed PI3K/Akt/mTOR signalling by activating PTEN (*p* = 0.005) and by dephosphorylating Akt (*p* = 0.002) and S6RP (*p* = 0.033); it also inhibited MAPK/ERK pathway by dephosphorylating c-Raf (*p* = 0.018) and ERK1/2 (*p* = 0.002). In ER-positive (MCF-7, T47D) and ER-negative (MDA-MB-231, BT-549) breast cancer cells, simvastatin treatment consistently induced apoptosis and inhibited proliferation by deregulating caspase cascades and cell cycle proteins in a dose dependent manner. Concordantly, simvastatin strongly suppressed PI3K/Akt/mTOR pathway by enhancing PTEN expression and by further sequentially dephosphorylating downstream cascades including Akt, mTOR, p70S6K, S6RP and 4E-BP1. Furthermore, simvastatin significantly inhibited MAPK/ERK pathway by dephosphorylating sequential cascades such as c-Raf, MEK1/2 and ERK1/2. These simvastatin anti-tumoral effects were reversed by metabolic products of the mevalonate pathway, including mevalonate, farnesyl pyrophosphate and geranylgeranyl pyrophosphate. These findings shed light on the biological and potential anti-tumoral effects of simvastatin in breast cancer.

## INTRODUCTION

Commonly used as cholesterol-lowering drugs, statins are small-molecule inhibitors of 3-hydroxy-3-methylglutaryl coenzyme A (HMG-CoA) reductase of the mevalonate pathway, also known as cholesterol biosynthesis pathway. Recently, statins have attracted attention as potential therapeutic agents in various types of cancer, including colon, lung and breast [1-3]. An epidemiological study in a Danish cohort showed a 15% reduction in all-cause and cancer-specific mortality in statins users as compared to non-users [4]. Preclinical studies have shown that statins play an important role in suppressing proliferation, inducing apoptosis and inhibiting metastasis of breast cancer [3, 5, 6]. In window-of-opportunity clinical trials in breast cancer, short term statins treatment exerted anti-tumoral effects by inhibiting proliferation and promoting apoptosis [7-9]. One study suggested the anti-proliferation effects of statins to be more significant in HMG-CoA reductase positive breast cancer [10].

As mevalonate pathway inhibitors, the potential mechanisms of anti-tumoral effects of statins are complex, including both cholesterol-dependent and -independent effects. Cholesterol-dependent effects include the deprivation of tumor cells from their increased demand of cholesterol uptake [11, 12]. On the other hand, cholesterol-independent pleiotropic effects exerted by statins may also contribute to their anti-tumoral effects [3, 13]. By suppressing HMG-CoA reductase, statins decrease levels of mevalonate and downstream lipid isoprenoid intermediates, such as farnesyl pyrophosphate (FPP) and geranylgeranyl pyrophosphate (GGPP) [14]. These isoprenoid intermediates provide lipid attachments for intracellular G-proteins, including Ras and Rho, which must undergo post-translational prenylation by FFP or GGPP to enable their translocation from cytoplasm to cell membrane [14]. Through inhibition of prenylation of Ras and Rho proteins, statins may suppress extensive downstream signalling pathways of these proteins, such as the PI3K/Akt/mTOR and MAPK/ERK pathways, which are commonly abrogated in many types of cancer. A recent global gene expression study has reported atorvastatin to inhibit the MAPK pathway in breast cancer biopsies [8]. However, the precise mechanisms by which statins suppress proliferation and induce apoptosis of breast cancer are still not fully understood. The biological alterations following statins treatment in prospective clinical trials, particularly their potential effects on PI3K/Akt/mTOR and MAPK/ERK pathways, are lacking.

In this study, immunohistochemistry (IHC) staining on breast cancer clinical samples treated with simvastatin prospectively and *in vitro* functional studies on breast cancer cell lines were applied with the following objectives: 1) to explore the effects of simvastatin on apoptosis, proliferation and Ras downstream pathways including PI3K/Akt/mTOR and MAPK/ERK in a window-of-opportunity breast cancer trial; 2) to further confirm findings from the clinical trial with functional studies using breast cancer cell lines; and 3) to explore the regulatory role of mevalonate pathway on the anti-tumoral effects of simvastatin. These findings shed light on the biological and potential therapeutic effects of simvastatin in breast cancer.

## RESULTS

### Simvastatin induced apoptosis and suppressed proliferation of breast cancer in clinical specimens (Figure 1A)

**Figure 1:**
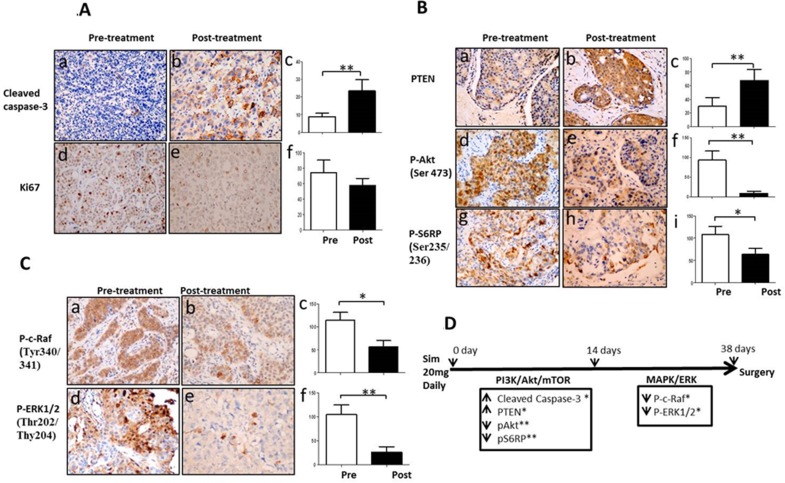
Simvastatin induced apoptosis, inhibited proliferation and deactivated PI3K/Akt/mTOR and MAPK/ERK pathways in a window-of-opportunity trial of breast cancer Immunohistochemistry staining was performed in paired pre- and post-treatment tumors with simvastatin. Magnification X200. Figure **1A:** Changes in expression of cleaved caspase-3 and Ki67. (a-c) cleaved caspase-3; (d-f) Ki67; Figure **1B**: Changes in activation of PI3K/Akt/mTOR pathway. (a-c) PTEN; (d-f) phospho-Akt (Ser473); (g-i) phospho-S6 ribosomal protein (Ser235/236); Figure **1C:** Changes in phosphorylation of MAPK/ERK pathway. (a-c) phospho-c-Raf (Tyr340/341); (d-f) phospho-ERK (Thr202/Tyr204). Figure **1D:** Schema of the time-points of apoptosis induction and dephosphorylation of PI3K/Akt/mTOR and MAPK/ERK pathway during up to 38 days of simvastatin treatment. * P < 0.05; ** P < 0.01.

15 female patients with newly diagnosed primary breast cancer received 5-38 days of simvastatin at a dose of 20 mg daily before definitive breast cancer surgery. Pre- and post-treatment tumor biopsies were obtained before starting simvastatin and at surgery, respectively. Significant induction of apoptosis as determined by positive cleaved caspase-3 was seen in post-treatment tumors (8.9±7.4 *vs* 23.4±24.3, *p* = 0.002). Decreased trend in Ki67 was observed in post-treatment in relation to pre-treatment samples, although p-value did not reach significance (74.6±59.9 *vs* 57.7±35.2, *p* = 0.245).

### Simvastatin deactivated PI3K/Akt/mTOR and MAPK/ERK pathways of breast cancer in clinical specimens (Figure 1B and 1C)

Simvastatin significantly inhibited PI3K/Akt/mTOR signalling pathway by increasing PTEN expression (30.0±46.6 *vs* 66.1±65.2, *p* = 0.005) and decreasing phosphorylation of both Akt at Ser473 and S6RP at Ser235/236 (93.0±87.4 *vs* 9.0±19.5, *p* = 0.002 for p-Akt; 108.6±67.7 *vs* 63.7±51.6, *p* = 0.033 for p-S6RP; Figure 1B). Similarly, simvastatin also deactivated MAPK/ERK pathway by dephosphorylating c-Raf at Tyr340/341 and ERK1/2 at Thr202/Thy204 (107.3±67.1 *vs* 57.3±51.8, *p* = 0.018 for p-c-Raf and 105.0±73.1 *vs* 26.6±42.6, *p* = 0.002 for p-ERK1/2; Figure 1C). These data indicate that simvastatin dually deactivates both PI3K/Akt/mTOR and MAPK/ERK signalling pathways in breast cancer.

### Simvastatin-induced apoptosis and suppression of PI3K/Akt/mTOR pathway were early events while deactivation of MAPK/ERK pathway was a late event in clinical specimens (Figure 1D)

To further determine time-related responses upon exposure to simvastatin, patients were divided into two subgroups using mean treatment duration of 14 days as cut-off: ≤14 day group (mean 9.4±3.7, range 5 to 14 days, *n* = 9); >14 day group (mean 21.5±9.0, range 15 to 38 days, *n* = 6). As shown in Figure 1D, in tumors exposed to simvastatin ≤14 days, simvastatin significantly increased apoptosis by enhancing cleaved caspase-3 expression (10.5±8.9 *vs* 18.5±16.6, *p* = 0.039) and deactivated PI3K/Akt/mTOR pathway by enhancing PTEN expression (16.3±27.7 *vs* 36.9±41.8, *p* = 0.030) and by dephosphorylating both Akt and S6RP (106.7±82.5 *vs* 12.8±24.1 for p-Akt, *p* = 0.006; 132.5±46.5 *vs* 56.3±39.6 for p-S6RP, *p* = 0.003). However, no significant changes in the MAPK/ERK pathway were observed following short exposure to simvastatin ≤14 days (*p* = 0.237 for p-c-Raf; *p* = 0.069 for p-ERK1/2). As for tumors exposed to simvastatin >14 days, deactivation of Akt pathway continued to be observed with enhanced PTEN expression (48.3±62.1 *vs* 105.0 ±73.7, *p* = 0.012). Interestingly, deactivation of MAPK/ERK pathway was now obvious through dephosphorylation of both c-Raf and ERK1/2 (113.2±52.0 *vs* 40.0±39.0, *p* = 0.023 for p-c-Raf; 118.3±82.3 *vs* 28.0±33.5, *p* = 0.011 for p-ERK1/2) with longer exposure to simvastatin. These data suggest that simvastatin treatment with mean duration 9.4 days is sufficient to deactivate PI3K/Akt/mTOR pathway and induce cancer cell apoptosis, while longer exposure with mean duration 21.5 days is required for deactivation of MAPK/ERK pathway in human breast cancer cells *in vivo*.

### Simvastatin induced apoptosis of breast cancer cell lines (Figure 2A and 2B)

**Figure 2:**
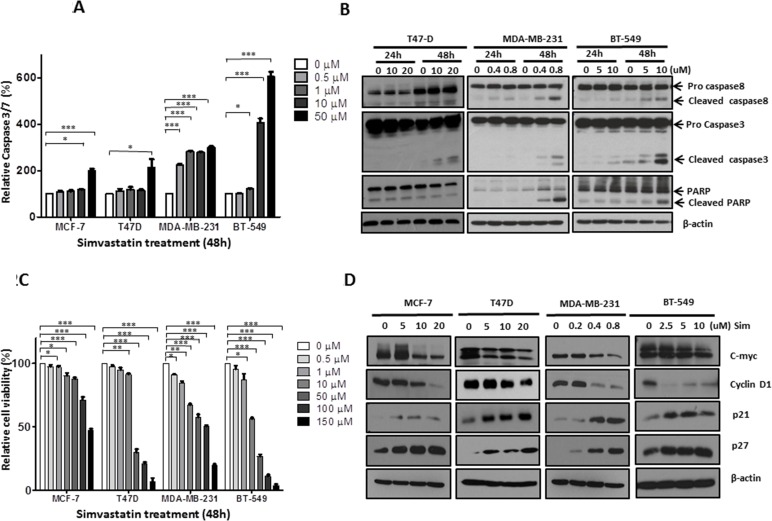
Simvastatin induced apoptosis and inhibited proliferation in breast cancer cells Figure **2A:** MCF-7, T47D, MDA-MB-231and BT-549 were exposed to simvastatin (range 0 to 50μM) for 48 hours and apoptosis was detected by measuring caspase-3/7 activity; Figure **2B:** After 24 and 48 hours treatment for T47D (dose from 0 to 20μM), MDA-MB-231 (dose from 0 to 0.8μM) and BT-549 (dose from 0 to 10μM), apoptosis were determined by Western bolt analysis with antibodies against caspase-8, caspase-3 and PARP; Figure **2C:** Cells were treated with simvastatin (range 0 to 150μM) for 48 hours and cell viability was measured by MTS assay; Figure **2D:** After 48 hours simvastatin treatment for MCF-7 (range 0 to 20μM), T47D (range 0 to 20μM), MDA-MB-231 (range 0 to 0.8μM) and BT-549 (range 0 to 10μM), proliferation was determined by Western blots by assessing the levels of c-myc, cyclin D, p21 and p27. ** P < 0.05; ** P < 0.01;*** P < 0.001.

Since we have observed the cytotoxic effects of simvastatin on breast cancer in clinical specimens, we then went on to explore its anti-cancer effects in ER-positive (MCF-7, T47D) and ER-negative (MDA-MB-231, BT-549) breast cancer cell lines. Simvastatin increased the activities of caspase-3/7 in a dose dependent manner following 48 hours treatment. 50μM simvastatin induced caspase-3/7 by 6-fold in BT-549, 3-fold in MDA-MB-231 and 2-fold in both MCF-7 and T47D (Figure 2A). MCF-7 cells are deficient in the expression of caspase-3 because of a spontaneous deletion within the CASP-3 gene [15], leading to proteolysis impairment of a range of caspase substrates in MCF-7 cells, including PARP [16]. Therefore, we focused on T47D, MDA-MB-231 and BT-549 cells for further validation on apoptosis. Using Western blot analysis, we confirmed significant cleavage of caspase-8, caspase-3 and PARP in cells treated for 48 hours, and obvious dose-dependent cleavages were seen at dosages as low as 0.4μM for MDA-MB-231 and 5μM for BT-549. As for T47D cells, significant cleavage of caspase-3 but not caspase-8 and PARP was found at dosage as low as 10μM following 48 hours treatment (Figure 2B), suggesting simvastatin-induced apoptosis was more caspase-3 dependent in a subpopulation of breast cancer cells. Collectively, apoptotic data suggest that simvastatin induces apoptosis through both enhancing the activities of caspase-3/7 and promoting the cleavage of caspase-8, caspase-3 and PARP in some breast cancer cells; caspase-3 appears to be a major caspase involved in simvastatin induced apoptosis. Previous reports also showed that lovastatin induced apoptosis by increasing activities of caspase cascades in other cancers [17, 18]. Therefore, caspases are identified as potential mediators of statins-induced apoptosis.

### Simvastatin inhibited proliferation of breast cancer cell lines (Figure 2C and 2D)

Similarly, we attempted to confirm the clinical observations of inhibitory effects of simvastatin on breast cancer growth by functional studies. MTS assay showed that simvastatin inhibited the growth of MCF-7, T47D, MDA-MB-231 and BT-549 in a dose dependent manner. Among these 4 cell lines, MDA-MB-231 appears to be the most sensitive, with significant inhibition of cell growth seen in cells treated at dosage as low as 0.5μM of simvastatin (Figure 2C). Western blot analysis further confirmed that simvastatin decreased the expression of c-myc and cyclin D1 and increased p21 and p27 in a dose dependent manner. Significant changes for these cell cycle biomarkers were seen at dosages as low as 10μM for both MCF-7 and T47D, 0.4 μM for MDA-MB-231 and 2.5μM for BT-549 (Figure 2D). Taken together, our findings suggest that simvastatin suppresses the expression of cell cycle-promoting proteins and increases cell cycle-suppressing proteins and thus inhibits cell cycle progression from G1 to S phase to suppress breast cancer cell proliferation.

### Simvastatin deactivated PI3K/Akt/mTOR pathway in breast cancer cell lines (Figure 3A)

**Figure 3:**
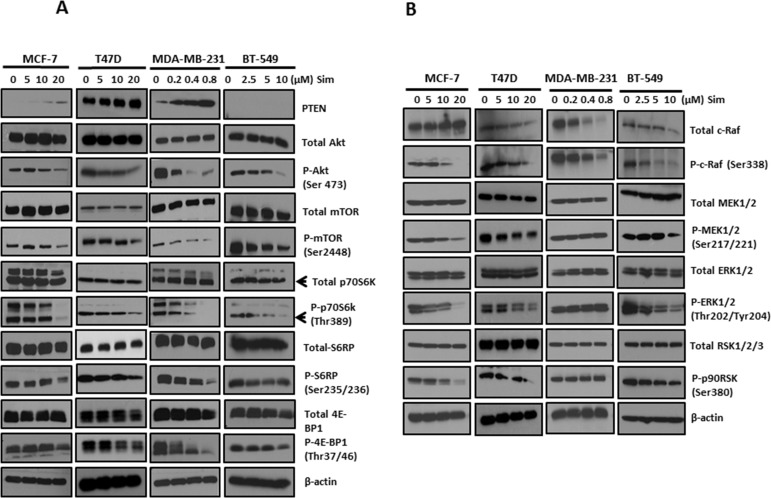
Simvastatin deactivated PI3K/Akt/mTOR and MAPK/ERK pathways in breast cancer cells Figure **3A**: The levels of phosphorylated and total proteins of PI3K/Akt/mTOR pathway, including PTEN, Akt, mTOR, p70S6K, S6RP and 4E-BP1, were assessed by Western blots following simvastatin treatment. Figure **3B:** Western blots were applied by assessing phosphorylation levels and total proteins of c-Raf, MEK, ERK and p90RSK to determine the activation of MAPK/ERK pathway in simvastatin-treated cells.

There is emerging evidence that constitutive activation of PI3K/Akt/mTOR pathway plays a critical role in the survival and growth of breast cancer cells. Here, we explored the effects of simvastatin on PI3K/Akt/mTOR pathway effectors, including PTEN, Akt, mTOR, p70S6K, S6RP and 4E-BP1. After 48 hours treatment, PTEN expression increased in MCF-7, T47D and MDA-MB-231 cells at dosages as low as 10μM for both MCF-7 and T47D and 0.2μM for MDA-MB-231 cells, respectively. PTEN expression is undetectable in BT-549 because it is a PTEN-null cell line [19]. It has been well-known that tumor suppressor PTEN dephosphorylates phosphatidylinositol-3,4,5-trisphosphate (PIP3) to phosphatidylinositol-4,5-bisphosphate (PIP2), thereby terminating PI3K-dependent signalling. Our findings suggest that, in PTEN-intact breast cancer cell lines, simvastatin suppresses PI3K pathway of breast cancer through enhancing PTEN expression. Furthermore, in all 4 cell lines, dose-dependent suppression of phosphorylation was noted for Akt at Ser473, mTOR at Ser 2488 and p70S6K at Thr389. These suggest that simvastatin is able to mediate other alternative effectors to inhibit Akt/mTOR signalling in PTEN-null tumors. Therefore, simvastatin treatment blocked PI3K downstream signalling through dephosphorylating Akt, mTOR and p70S6K. Moreover, we examined the effects of simvastatin on phosphorylation of S6 ribosomal protein, a direct target for p70S6K, whose phosphorylation promotes the translation of mRNA that has oligopyrimidine tracts in the 5′ untranslated region. Simvastatin treatment strongly suppressed phosphorylation of S6 ribosomal protein. We also examined the effects of simvastatin treatment on phosphorylation of the translational repressor 4E-BP1, a protein that plays a key regulatory role on the inhibition of cap-dependent mRNA translation by negatively controlling the function of elF4E. Simvastatin treatment of cells significantly suppressed phosphorylation of 4E-BP1 at Thr37/36. Thus, simvastatin appears to negatively regulate activation of PI3K/Akt and further block mTOR→S6 ribosomal protein and →4E-BP1 signalling cascades in breast cancer cells.

### Simvastatin suppressed MAPK/ERK pathway in breast cancer cells (Figure 3B)

To determine the effects of simvastatin on MAPK/ERK pathway, phosphorylation of c-Raf at Ser338, MEK1/2 at Ser217/221, ERK1/2 at Thr202/Tyr204 and p90RSK at Ser380 was assessed by Western blot analysis. 10 and 20μM simvastatin were required to dephosphorylate c-Raf in MCF-7 and T47D respectively, while a much lower concentration of 0.2 and 2.5μM simvastatin started to suppress c-Raf phosphorylation in MDA-MB-231 and BT-549, respectively. In addition, dose-dependent suppression of c-Raf expression was seen in both MDA-MB-231 and BT-549. These data suggest that c-Raf is simvastatin-sensitive in breast cancer cells. To further examine if downstream effectors of c-Raf would be inhibited by simvastatin, phosphorylation levels of MEK1/2, ERK1/2 and p90RSK were assessed by immunoblotting. Consistently, dephosphorylation of downstream effectors of c-Raf, MEK1/2, ERK1/2 and p90RSK, was seen in MCF-7, T47D and BT-549 treated with equal to or more than 10μM simvastatin for MCF-7 and T47D as well as 2.5μM for BT-549, suggesting simvastatin sequentially deactivates MAPK/ERK pathway in these cell lines. Surprisingly, simvastatin did not show similar effects on these c-Raf downstream effectors in MBA-MD-231. MDA-MB-231 harbours BRAF (G464V) mutation, which leads to constitutive activation of MAPK pathway, and this may have resulted in relative resistance to inhibition of this pathway by simvastatin. Previous reports on other cancers also confirmed the disruption of ERK1/2 phosphorylation by statins (32, 33). These data suggest that MAPK/ERK is another target pathway of simvastatin in cancer, which can explain, at least partially, the apoptosis induction and anti-proliferation effects of statins.

### Mevalonate pathway contributed to simvastatin induced apoptosis, proliferation inhibition and deactivation of PI3K/Akt and MAPK/ERK pathways in breast cancer cells (Figure 4)

**Figure 4:**
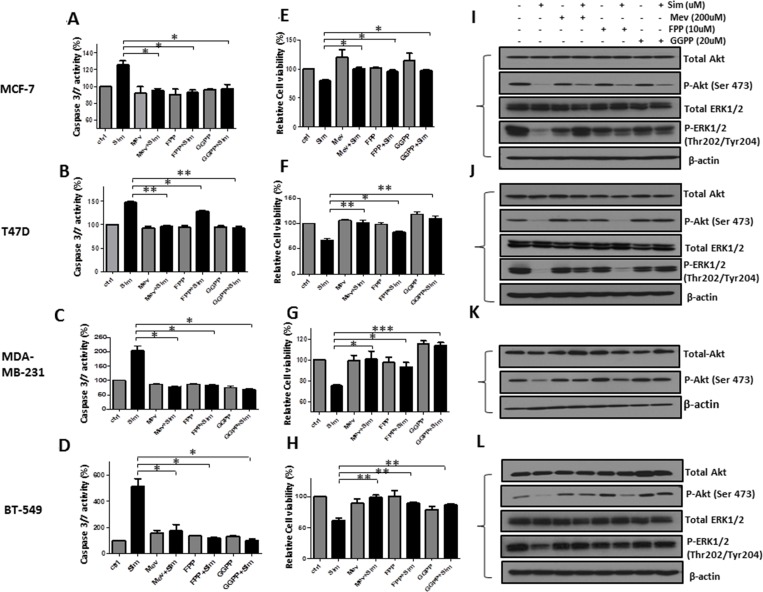
Simvastatin-induced anti-tumoral effects of apoptosis induction, proliferation inhibition and deactivation of PI3K/Akt and MAPK/ERK pathway were blocked by mevalonate, FPP and GGPP MCF-7, T47D, MDA-MD-231 and BT-549 were pre-treated with mevalonate (200μM), FPP (10μM) and GGPP (20μM) for 1 hour respectively, followed by incubation with or without simvastatin (20μM for MCF-7 and T47D; 0.8μM for MDA-MB-231 and 10μM for BT-549) for 48 hours. Figure **4A-D.** The activity of caspase-3/7 was determined by caspase-3/7 assay. Figure **4E-H.** Cell viability was determined by MTS assay. Figure **4I-L**. The levels of phosphorylated and total proteins of Akt and ERK1/2 were examined by Western blots analysis. * P < 0.05, ** P < 0.01 and ***P < 0.001 versus cells treated with simvastatin.

To further determine whether the anti-cancer effects of simvastatin is mediated by the blocking of mevalonate pathway, we performed add-back experiments using mevalonate, FPP or GGPP, intermediates of the mevalonate pathway. MCF-7, T47D, MDA-MB-231 and BT-549 cells were pre-treated with mevalonate, FPP or GGPP, followed by simvastatin treatment for 48 hours. Mevalonate, FPP and GGPP could reverse the levels of caspase-3/7 close to that in vehicle controls for MCF-7, MDA-MB-231 and BT-549 cells (*P* < 0.05 for mevalonate, FPP and GGPP versus simvastatin treatment respectively, Figure 4A, 4C-4D). However, in T47D cells, FPP only partially rescued the levels of caspase-3/7 compared to relatively full recovery with mevalonate and GGPP (Figure 4B). Similarly, MTS assay showed that mevalonate, FPP and GGPP were able to reverse the anti-cancer effects induced by simvastatin (*P* < 0.05 for mevalonate, FPP and GGPP versus simvastatin treatment respectively, Figure 4E-4H) in all 4 cell lines, although the rescue was again less complete with FFP for T47D cells. Moreover, Western blot analysis showed that simvastatin-induced dephosphorylation of Akt at Ser473 and ERK1/2 at Thr202/Tyr204 was markedly blocked by mevalonate, FPP and GGPP respectively in MCF-7 (Figure 4I), MDA-MB-231 (Figure 4K) and BT-549 (Figure 4L) cells; the reversal effects on phospho-Akt were incomplete compared to its relatively full recovery on phospho-ERK1/2. Our findings indicate that both protein farnesylation and geranylgeranylation play an important role in inhibiting PI3K/Akt and MAPK/ERK pathway by simvastatin in MCF-7, MDA-MB-231, and BT-549. However, in T47D cells (Figure 4J), the protective effects of mevalonate and GGPP on Akt and ERK1/2 phosphorylation were again more obvious than that of FPP, suggesting that deactivation of these two signalling pathways by simvastatin may be mainly regulated by inhibiting protein geranylgeranylation in T47D cells. Taken together, depletion of mevalonate, FPP and GGPP by simvastatin contributes to simvastatin-induced apoptosis, proliferation inhibition and deactivation of PI3K/Akt and MAPK/ERK pathways in breast cancer cell lines.

## DISCUSSION

Observational studies and meta-analyses have demonstrated that statins exposure could cause reduction in risk of multiple cancers, including breast cancer [4, 20-22]. Preclinical reports have also shown that statins play important roles in inducing apoptosis and suppressing proliferation of breast cancer [3, 5, 6]. In this study, clinical samples treated prospectively together with *in vitro* functional studies concordantly show simvastatin to induce apoptosis, suppress proliferation and dephosphorylate sequential signalling cascades of PI3K/Akt/mTOR and MAPK/ERK pathways of breast cancer. These simvastatin-induced anti-tumoral effects are regulated by suppression of mevalonate pathway. To our best knowledge, the current study is the first prospective clinical report, which shows simvastatin-induced breast cancer death and suppression of both PI3K/Akt/mTOR and MAPK/ERK pathways.

Previous studies have drawn our attention to the relatively higher sensitivity to statins of ER-negative breast cancer cells [23-25]. Although our data from clinical specimens were not able to show the differential response to simvastatin between ER-positive and ER-negative tumors probably due to the relatively small sample size of this clinical study (*n* = 3 for ER-negative patients), functional studies concordantly showed ER-negative breast cancer cells (MDA-MB-231 and BT-549) to be more sensitive than ER-positive cells (MCF-7 and T47D) to simvastatin treatment. Significant biological effects were found at relatively lower simvastatin doses for ER-negative breast cancer cells than ER-positive ones. A previous study showed estrogen protected ER-positive breast cancer from the anti-proliferative effects of statins [26], and this may in part explain the differential sensitivity to statins between ER-negative and ER-positive breast cancers. The exact mechanisms which confer relative resistance of ER-positive breast cancer to statins warrant further study.

The presence of concurrent multiple activated signalling pathways contributes to the heterogeneous nature of breast cancer and thus necessitates multi-targeted approaches for effective breast cancer prevention and therapy. In breast cancer, constitutive activation of PI3K/Akt/mTOR and MAPK/ERK signal pathways is an important event, which regulates multiple cellular processes to promote cancer cell growth, survival, and metastasis [27, 28]. An emerging concept is targeting two or more different signal transduction pathways as a therapeutic strategy, for example, PI3K/Akt/mTOR and MAPK/ERK [29]. This has been explored in some preclinical models as well as clinical trials. Previous preclinical studies showed that dual inhibition of PI3K/Akt/mTOR and MAPK/ERK pathways by combining two different small molecule kinase inhibitors led to more efficient growth inhibition than single pathway inhibition in both breast and lung cancers [26, 30]. Strikingly, a recent phase I clinical trial in patients with advanced cancers confirmed that dual blockade of both PI3K/AKT/mTOR and MEK/ERK pathways resulted in more favourable efficacy compared to single blockade of either pathway [31]. This is probably due to the interconnection between PI3K and MAPK pathway with multiple points of convergence, cross-talk, and feedback loops. For example, ERK1/2 converges on mTORC1 via P90RSK, leading to nuclear transcriptional changes that regulate cell proliferation and survival [32]. Signalling can remain active by alternative pathways despite inhibition of one pathway, making concurrent inhibition of both PI3K and MAPK pathway a rational therapeutic approach. However, dual blockade of both PI3K/AKT/mTOR and MEK/ERK pathways by combining small molecule kinase inhibitors were usually achieved at the expense of higher toxicity and financial cost [31]. Developing a safe therapeutic agent that has multiple targets is thus an attractive alternative. In this study, we found simvastatin to induce apoptosis, inhibit proliferation of breast cancer and deactivate sequential cascades of both PI3K/Akt/mTOR and MAPK/ERK pathways. Our data on MAPK pathway inhibition by simvastatin is consistent with a recent gene expression study on clinical specimens of breast cancer treated with statins [8]. Simvastatin is cheap, safe, and well-tolerated, with a long track record of clinical use in the treatment of hypercholesterolemia. Thus, simvastatin has the potential to be used as a multi-targeted agent to inhibit both PI3K/AKT/mTOR and MEK/ERK signalling and consequently suppress tumour growth. Moreover, aberrant activation of both PI3K/Akt/mTOR and MAPK/ERK signalling pathways has been associated with resistance to conventional therapy in breast cancer, including both chemotherapy and endocrine therapy [33-35]. Therefore, the combination of statins and chemotherapy or endocrine therapy is a promising strategy to overcome resistance to conventional therapy in breast cancer, which warrants further investigation.

Interestingly, in clinical specimens, we found early effects on PI3K/Akt/mTOR signalling and relatively later effects on MAPK/ERK signalling in response to simvastatin (mean duration exposure 9.4 *vs* 21.5 days). Although data on the differential responses between PI3K/Akt/mTOR and MAPK/ERK signalling following statins treatment in cancer are very limited, sensitivity to inhibitors of these two signalling pathways has been linked to genetic mutations in key element in these pathways, such as PIK3CA, PTEN, Akt and Raf [36, 37]. In breast cancer, mutations in individual components in the PI3K/Akt/mTOR pathway are more frequent than in the MAPK/ERK pathway [38], and this may be one reason why earlier effects were seen in the PI3K/Akt/mTOR pathway relative to the MAPK/ERK pathway. We acknowledge that our observation of differential time-based response of PI3K/Akt/mTOR and MAPK/ERK is on a relatively small number of patients (*n* = 15), and tumor biopsies collected at early and late time-points were not obtained from the same patients; further prospective study with collection of serial tumor biopsies (*e.g.*, pretreatment, 9-days and 21-days post-treatment) from the same patients of a large-sized cohort is warranted to further confirm the current findings.

Mevalonate pathway, also defined as cholesterol synthesis pathway, plays an important role in breast carcinogenesis, growth and metastasis. Exogenous expression of HMG-CoA reductase deregulated the mevalonate pathway and facilitated transformation in human breast cancer cell lines [39]. 27HC, a primary metabolite of cholesterol, promoted tumor growth and metastasis in mouse models of breast cancer [40]. Thus, blocking mevalonate pathway may be a useful strategy for breast cancer prevention or treatment. Through inhibiting HMG-CoA reductase in the mevalonate pathway, simvastatin suppresses the generation of mevalonate, FPP and GGPP and further deregulates both cholesterol biosynthesis and protein prenylation [14]. In cancer treatment, statins are believed to serve as prenylation inhibitors of Ras GTPase, which may suppress extensive Ras downstream signalling pathways, such as PI3K/Akt/mTOR and MAPK/ERK [14, 41]. Consistent with previous findings in lymphoma [42] and colorectal cancer [43], we showed that individual add-back with mevalonate, FPP and GGPP was able to reverse simvastatin-induced apoptosis, proliferation inhibition and deactivation of PI3K/Akt and MAPK/ERK signalling in breast cancer cell lines. Strikingly, we found that mevalonate, FPP and GGPP were able to rescue the dephosphorylation of both Akt and ERK1/2 by simvastatin in MCF-7, MDA-MB-231 and BT-549, whereas only mevalonate and GGPP but not FPP reversed the deactivation of Akt and ERK1/2 in T47D (Figure 4J). Our findings indicate that depletion of farnesylated- and geranylgeranylated-protein by simvastatin deactivates PI3K/Akt and MAPK/ERK pathway in MCF-7, MDA-MB-231 and BT-549. However, in T47D cells, suppression of PI3K/Akt and MAPK/ERK may be more dependent on inhibition of protein geranylgeranylation by simvastatin. The crucial prenylated proteins inhibited by simvastatin in breast cancer are still unknown. Further studies to identify the specific prenylated proteins that are modified by simvastatin will help to design improved simvastatin-based therapeutic strategies. Moreover, we found the reversal effects on phospho-Akt at Ser473 by adding metabolic products to be incomplete compared to the relatively full recovery on phospho-ERK1/2 at Thr202/Tyr204 in MCF-7 (Figure 4I), MDA-MB-231 (Figure 4K) and BT-549 (Figure 4L) cells. This indicates that simvastatin may additionally suppress PI3K/Akt pathway through mevalonate-independent mechanisms. Recently, accumulating evidence suggests that cholesterol plays an important role in cancer progression and development through activating survival signalling of Akt [44-46]. Further exploration on the effects of simvastatin on the levels of intracellular cholesterol in breast cancer may help to explain these current findings.

Simvastatin is not the most potent statin in terms of reducing the levels of low density lipoprotein-cholesterol. Dose-for-dose, rosuvastatin and atorvastatin are more effective than simvastatin [47]. Despite this, simvastatin is observed to exert systemic anti-tumoral effects in our window-of-opportunity clinical trial. To date, simvastatin has shown the most data on potential anti-cancer effect in both pre-clinical and clinical studies, in part because it is one of the most widely prescribed statins [48-52]. Simvastatin certainly warrants exploration as a therapeutic agent in breast cancer. To further exploit statins in cancer therapeutics, it will also be worthy to study other more potent statins, such as rosuvastatin and atorvastatin, in breast cancer therapeutics.

In this study, breast cancer clinical samples treated prospectively, together with *in vitro* functional studies, concordantly show simvastatin to induce apoptosis, inhibit proliferation and suppress PI3K/Akt/mTOR and MAPK/ERK pathways in breast cancer, and these anti-tumoral effects are achieved by inhibiting the mevalonate pathway. Our findings provide the basis for future clinical trials design. Prospective trials which incorporate simvastatin together with conventional treatments, such as chemotherapy and endocrine therapy, will be interesting to directly address if incorporating statins in anti-cancer therapy can indeed improve treatment efficacy in breast cancer patients.

## PATIENTS, MATERIALS AND METHODS

### Patients

A prospective window-of-opportunity trial was conducted at the National University Cancer Institute, Singapore; 15 female patients with newly diagnosed primary breast cancer received 5-38 days of simvastatin at a dose of 20 mg daily before definitive breast cancer surgery. Pre- and post-treatment tumor biopsies were obtained before starting simvastatin and at surgery, respectively. The clinicopathological characteristics of the patient cohort were summarized in Table 1. This study was approved by the institutional ethics review board and written informed consent was obtained from all patients.

**Table 1:** Clinicopathological characteristics of breast cancer patients enrolled into a window-of-opportunity study with simvastatin

Parameters		N (%)
Age	Median age 52.7; range 41 to 70	
Race	Chinese	6 (40.0)
	Malay	7 (46.7)
	Indian	1 (6.7)
	Others	1 (6.7)
Histological tumor type	Invasive ductal carcinoma	11 (73.3)
	Invasive lobular carcinoma	2 (13.3)
	Others	2 (13.3)
Tumor grade	2	8 (53.3)
	3	7 (46.7)
Estrogen Receptor	Negative	3 (20.0)
	Positive	12 (80.0)
Progesterone Receptor	Negative	3 (20.0)
	Positive	12 (80.0)
Human Epidermal Growth Factor Receptor 2	Negative	13 (86.7)
	Positive	2 (13.3)

### IHC staining

As described previously [53-55], consecutive sections of 4μm thickness were cut from pre- and post-treatment tumor samples. Slides were stained with a BondMax Autostainer (Leica, Wetzlar, Germany) according to the instructions of the manufacturer. Antibodies used for IHC staining were listed in Supplementary Table 1.

### Scoring of IHC expression

The expression of cleaved caspase-3, PTEN, p-Akt, p-S6RP, p-c-Raf and p-ERK1/2 in tumor cells was scored by H-score [53, 54]. Briefly, immunoreactivity of biomarkers was scored by multiplying the intensity and extent of staining from the same specimen according to the following formula:

IHC score=staining intensity×staining extent

IHC score range was from 0 to 300. Staining intensity was classified into four groups: 0 (negative), 1 (weak), 2 (moderate), and 3 (strong). Staining extent was defined as the approximate percentage of positive staining cells in the total cancer compartment. Ki67 proliferation index was assessed by calculating Ki67 positive tumor nuclei in 500 tumor cells [10].

### Cell lines and reagents

Human breast cancer cell lines MCF-7 (ER+), T47D (ER+), MDA-MB-231 (ER-) and BT-549 (ER-) were purchased from American Type Culture Collection (ATCC, Manassas, VA, USA). Cells were maintained in high glucose Dulbecco's Modified Eagle's Medium (MCF-7 and MDA-MB-231) or RPMI 1460 media (T47D and BT-549) (Nacalai Tesque, Japan), supplemented with 10% fetal bovine serum and 1X Penicillin-Streptomycin (Invitrogen, Carlsbad, CA) at 37°C in a humidified atmosphere with 5% CO_2_. Cell lines were re-authenticated upon receipt. Simvastatin, mevalonate, ammonium salt of FPP and GGPP were purchased from Sigma-Aldrich Co (St. Louis, MO, USA). Simvastatin was dissolved in DMSO which was used as vehicle control. Antibodies used for Western blot analysis were listed in Supplementary Table 1.

### Cell treatment

Cells were seeded in 96-well plates or 10cm dishes and treated with simvastatin at the indicated concentrations for 24 or 48 hours. To determine if metabolic products of the mevalonate pathway could block the anti-tumoral effects of simvastatin, cells were pre-treated with 200μM mevalonate, 10μM FPP and 20μM GGPP respectively for 1 hour and then incubated with or without simvastatin for 48 hours, at dosage showing significant anti-tumoral effects on breast cancer cells (20μM for MCF-7 and T47D; 0.8μM for MDA-231; 10μM for BT-549). All the experiments were performed in three independent assays.

### Caspase-3/7 assay

Cells treated in 96-well plates were mixed with Caspase-3/7 Reagent (Promega, Madison, WI) according to the instructions of the manufacturer and caspase-3/7 activities were assessed using a plate-reading luminometer (Tecan, Männedorf, Switzerland).

### Cell viability assay

Cells treated in 96-well plates were mixed with CellTiter 96 Aqueous One Solution Reagent containing a tetrazolium compound [3-(4,5-dimethylthiazol-2-yl)-5-(3-carboxymethoxyphenyl)-2-(4-sulfophenyl)-2H-tetrazolium, inner salt; MTS] (Promega, Madison, WI) according to the instructions of the manufacturer and the absorbance at 490nm was determined using a 96-well plate reader (Tecan, Männedorf, Switzerland).

### Western blot analysis

After treatment with simvastatin at the indicated concentrations, cell pellets were collected and lysed. Proteins were separated by SDS polyacrylamide gel electrophoresis, transferred to polyvinylidene fluoride (PVDF) membranes (Millipore, Billerica, MA), incubated with different primary antibodies and detected by enhanced chemiluminescent immunodetection system (GE Healthcare Life Science, Little Chalfont, UK).

### Statistical analysis

The different immunoreactivity expression of biomarkers between pre- and post-treatment tumors was analyzed using paired *t*-test. The anti-tumoral effects of simvastatin on breast cancer cells compared with vehicle controls, as well as the protective effects of mevalonate, FPP and GGPP on breast cancer cells, were determined with *t-*tests. Statistical analyses were performed using IBM SPSS package (version 19.0 for Windows, IBM SPSS Inc., USA) with significance at 5%.

## SUPPLEMENTARY MATERIAL TABLE


