# Supplementary material for: Simvastatin-induced breast cancer cell death and deactivation of PI3K/Akt and MAPK/ERK signalling are reversed by metabolic products of the mevalonate pathway
Source: Oncotarget. 2015 Nov 11;7(3):2532–44. doi: 10.18632/oncotarget.6304 (PMC4823053; doi:10.18632/oncotarget.6304)
Supplement: Supplementary file 1 [file oncotarget-07-2532-s001.pdf]

Simvastatin-induced breast cancer cell death and deactivation of PI3K/Akt and MAPK/ERK signalling are reversed by metabolic products of the mevalonate pathway

Supplementary Material

Supplementary table 1: Antibodies used for immunohistochemstry (IHC) staining and Western blots analysis

Purposes	Antibodies	Manufacturers
IHC	cleaved caspase-3	Cell Signaling Technology
	ki67	Merck Millipore
	PTEN	Cell Signaling Technology
	p-Akt (Ser473)	Cell Signaling Technology
	p-S6 ribosomal protein (Ser235/236)	Cell Signaling Technology
	p-c-Raf (Tyr340/341)	Santa Cruz Biotechnology
Western blots	p-ERK1/2 (Thr202/Thy204)	Cell Signaling Technology
	caspase-8	Cell Signaling Technology
	casepase-3	Cell Signaling Technology
	PARP	Cell Signaling Technology
	c-myc	Cell Signaling Technology
	cyclin D1	Cell Signaling Technology
	p21	Cell Signaling Technology
	p27	Cell Signaling Technology
	PTEN	Cell Signaling Technology
	p-PTEN (Ser380/Thr382/383)	Cell Signaling Technology
	Akt	Cell Signaling Technology
	p-Akt (Ser473)	Cell Signaling Technology
	mTOR	Cell Signaling Technology
	p-mTOR (Ser2448)	Cell Signaling Technology
	p70S6K	Cell Signaling Technology
	p-p70S6K (Thr389)	Cell Signaling Technology
	S6 ribosomal protein (S6RP)	Cell Signaling Technology
	p-S6RP (Ser235/236)	Cell Signaling Technology
	4E-BP1	Cell Signaling Technology
	p-4E-BP1 (Thr37/36)	Cell Signaling Technology
	c-Raf	Cell Signaling Technology
	p-c-Raf (Ser338)	Cell Signaling Technology
	MEK1/2	Cell Signaling Technology
	p-MEK1/2 (Ser217/221)	Cell Signaling Technology
	ERK1/2	Cell Signaling Technology
	p-ERK1/2 (Thr202/Tyr204)	Cell Signaling Technology
	p90 ribosomal S6 kinases (RSK1-3)	Cell Signaling Technology
	p-p90RSK(Ser380)	Cell Signaling Technology
	β-actin	Cell Signaling Technology
